# Evaluation of the Hologic Aptima Vaginitis assays for microbiological diagnosis of vaginitis in women with abnormal vaginal discharge attending primary care

**DOI:** 10.1099/jmm.0.002163

**Published:** 2026-05-19

**Authors:** Barry Vipond, Nicola Childs, Rich Hopes, David Hirst, Kshitij Soni, Paul North, Peter Muir

**Affiliations:** 1 UK Health Security Agency Clinical Network Laboratory, Southmead Hospital, Bristol, UK; 2 North Bristol NHS Trust, Southmead Hospital, Bristol, UK

**Keywords:** Aptima BV, Aptima CV/TV, bacterial vaginosis, *Candida*, diagnostic accuracy study, vaginitis

## Abstract

*An erratum of this article has been published full details can be found at* *https://doi.org/10.1099/jmm.0.002185.*

**Introduction.** Infectious vaginitis, caused mostly by bacterial vaginosis (BV), *Candida* vaginitis (CV) and *Trichomonas* vaginitis (TV), is a common reason for primary care visits. Recent studies demonstrate the potential utility of molecular assays in replacing conventional diagnostic methods.

**Hypothesis/Gap Statement.** Different diagnostic standards are in use for BV; published studies have evaluated molecular diagnostic tests against Amsel criteria, Nugent score or a combination of both. In the UK, Hay/Ison criteria are commonly used, but there are limited studies evaluating molecular diagnostic assays against these criteria.

**Aim.** This study evaluated the performance of Hologic’s Aptima® BV and CV/TV assays relative to Hay/Ison criteria for BV and culture for CV in women with symptoms of vaginitis attending primary care centres in the UK.

**Methodology.** Paired vaginal swabs were collected from women ≥18 years of age undergoing testing for *Chlamydia trachomatis* and *Neisseria gonorrhoea* using the Hologic Aptima Combo 2 (AC2) assay, alongside standard of care (SOC) testing for vaginitis (Hay/Ison criteria for BV; culture for CV). Samples received for AC2 testing were tested for vaginitis using Hologic Aptima BV and Aptima CV/TV assays. Sensitivity, specificity, positive and negative predictive values and diagnostic accuracy of Aptima BV and Aptima CV/TV assays relative to SOC methodology were determined. For discrepancy analysis, selected samples were evaluated further using third-party (Thermo Fisher) Vaginal Plus and pan-Candida real-time PCR.

**Results.** This study included 2,152 women. The mean (standard deviation) age of participants was 35.1 (11.5) years. Sensitivity and specificity estimates for the Aptima BV assay were 98.8% and 77.7% and 94.9% and 88.3% for CV detection by Aptima CV/TV, respectively, relative to SOC. Overall diagnostic accuracy values were 80.2% for Aptima BV and 90.0% for Aptima CV/TV. Although not routinely tested for in our population, a limited evaluation of TV detection by the combined Aptima CV/TV assay relative to the monoplex Aptima TV assay in 75 women indicated sensitivity and specificity values of 87.5% and 100%, respectively.

**Conclusion.** The Aptima BV and CV/TV assays demonstrated high sensitivity and specificity relative to SOC methodology in UK laboratories and are a viable alternative to conventional vaginitis testing.

**Impact Statement**

A number of scoring systems are in use to diagnose bacterial vaginosis (BV), including Nugent scoring, Amsel criteria and Hay/Ison criteria (described in the ‘Introduction’ section). A small number of studies have evaluated the performance of Aptima Vaginitis assays, comparing Aptima BV results with Nugent scoring, with or without Amsel criteria for resolution of indeterminate results. Hay/Ison criteria is a simplified scoring system recommended for use in sexual health clinics and is also widely used in primary care. This study is, to our knowledge, the first reported evaluation of the Aptima BV assay relative to Hay/Ison criteria and, as such, provides useful information in addition to that already published when considering a change from microscopic to molecular diagnostic testing. In addition, we have undertaken detailed discrepancy analysis to understand the microbiological basis of intermediate BV microscopy and discrepancies between Aptima Vaginitis and BV microscopy and *Candida* culture; this could also support decision-making in considering a change in test methodology.

## Data Summary

Raw data are depicted in screen captures provided in the accompanying supplement. Requests for any further information will be considered upon reasonable request to the corresponding author. The primary dataset used for this study will not be made publicly available because we do not have consent to share individual patient data.

## Introduction

Vaginitis, characterized by vaginal discharge, odour, irritation and pruritus [1], is a common reason for gynaecologic primary care visits [2]. The three main infective causes of vaginitis are bacterial vaginosis (BV) (40–50% of cases), *Candida* vaginitis (CV) (20–25%) and *Trichomonas* vaginitis (TV) (15–20%). Non-infectious causes, including atrophic, irritant, allergic and inflammatory vaginitis, account for 5–10% of cases [3].

BV occurs due to a shift in composition of the vaginal microflora from the normally dominant *Lactobacillus* species to anaerobe-dominated microbiota, including *Gardnerella*, *Atopobium*, *Mobiluncus*, *Prevotella*, *Bacteroides* and *Peptostreptococcus* species [4,6]. CV is defined as vaginal inflammation in the presence of *Candida* species, most commonly *Candida albicans*, followed by *Candida glabrata*, and the absence of other infectious agents [7]. The anaerobic protozoan parasite *Trichomonas vaginalis* is the cause of TV [1].

If left untreated, vaginitis can lead to severe complications, including pelvic inflammatory disease, which is associated with chronic pelvic pain, ectopic pregnancy and infertility [8]. Vaginitis also disrupts the vaginal mucosal barrier and immune defences, increasing the risk of contracting and transmitting sexually transmitted infections [8,9]. In pregnancy, BV and TV are associated with pre-term labour, low birth weight and amniotic fluid infection, posing risks to both the mother and baby [8,9]. Thus, early diagnosis is critical for guiding appropriate treatment. However, the common symptomatology of BV, CV and TV [3,10], the incidence of mixed infections or coinfections [11,13] and the recurrence of vaginal symptoms [14,17] make accurate diagnosis challenging.

Various diagnostic methods are available to identify the underlying cause of vaginitis. For BV, diagnosis often relies on Amsel’s criteria, involving the presence of three out of four of the following characteristics: (i) a thin, watery homogenous discharge; (ii) elevated vaginal pH (>4.5); (iii) a ‘fishy’ smell that is either spontaneously present or is generated by the addition of 10% potassium hydroxide to vaginal secretions; and (iv) microscopic examination revealing 20% vaginal clue cells [18,20]. However, the Amsel method is often impractical in a primary care setting; therefore, many physicians prefer Nugent scoring of a vaginal Gram smear [21], for which test results are presented as a score ranging from 0 to 10 (0–3, normal flora; 4–6, intermediate or mixed flora; 7–10, BV-positive). This is considered the ‘gold standard’ laboratory-based diagnostic method for BV.

The Hay/Ison criteria is an alternative scoring system [22], in which the vaginal microflora is divided into three categories: grade 1 (normal: *Lactobacillus* morphotypes predominate), grade 2 (intermediate: mixed flora with *Lactobacillus* and *Gardnerella* and/or *Mobiluncus* morphotypes) and grade 3 (BV-positive: predominantly *Gardnerella* and/or *Mobiluncus* morphotypes, with few or absent *Lactobacillus* morphotypes) [23]. While not necessarily considered the gold standard, the Hay/Ison criteria offer a simpler, more intuitive scoring system for clinical settings, which may make it more reproducible among non-expert microscopists. As such, the UK Standards for Microbiological Investigation currently recommend using either Nugent or Hay/Ison criteria for the investigation of BV [24]. For CV, there is currently no consensus on a gold standard diagnostic method, with some guidelines recommending microscopy, whereas others advise yeast culture [18,25,27].

Despite their routine use in clinics, microscopy and culture-based methods for the diagnosis of infectious vaginitis are labour-intensive, poorly standardized, and the interpretation of slide smears is a subjective process [28]. Microscopy presents challenges in terms of the level of expertise required to correctly interpret Gram smears, while the ‘intermediate’ category utilized with the standard of care (SOC) for BV introduces diagnostic uncertainty and the need for consensus scoring [29]. Routine culture for CV is also resource-intensive and often limited by variable sensitivity and inconsistency of species-level identification [30,32]. Traditional diagnostic methods for TV, such as wet mount microscopy, are also impractical in most routine workflows, as they require sample processing within 10 min in order to visualize live, motile *T. vaginalis* organisms [33]. TV culture also lacks sensitivity due to the loss of viability during specimen transit. As such, current British Association for Sexual Health and HIV guidelines recommend nucleic acid amplification testing as the preferred diagnostic approach for TV, supporting the shift toward molecular assays in clinical practice [34].

Indeed, recent studies have demonstrated the broader clinical utility of molecular diagnostic tests for the most common causes of infectious vaginitis, with an increasing number of commercially available molecular assays achieving a high degree of sensitivity and specificity, compared with SOC diagnostic methods (reviewed in references 30–32). These include the BD Affirm III (Beckton Dickinson), which uses DNA hybridization for diagnosis of BV, CV and TV [35,36]; the BD MAX Vaginal Panel (Beckton Dickinson), which uses multiplex quantitative PCR to diagnose BV, CV and TV [36,37]; the Aptima^®^ BV and Aptima^®^ CV/TV assays (Hologic), which together form the Aptima Vaginitis panel and use multiplex, real-time, transcription-mediated amplification to detect and quantify ribosomal RNA from indicator microorganisms that characterize the presence or absence of BV, CV and TV [29,33, 38, 39]; the Allplex Vaginitis assay (Seegene), which uses multiplex real-time PCR to diagnose BV, CV and TV [40]; the Xpert Xpress Multiplex Vaginal Panel (Cepheid), which uses real-time PCR to diagnose BV, CV and TV [41]; and the Cobas BV/CV (Roche), which uses real-time PCR to diagnose BV and CV. These commercial assays can be used in different settings, ranging from point-of-care testing to high-throughput laboratory testing.

There are limited publications comparing different molecular assays [35,36] or comparing molecular tests to SOC methods. However, there are several publications comparing Aptima BV with SOC in women with suspected BV, where diagnostic accuracy studies carried out in Canada [29], France [38], New Zealand [39] and the USA [33] have reported the Aptima BV assay to have a sensitivity of 90.0–98.4% and specificity of 89.6–96.3%, relative to Nugent score (excluding samples with intermediate microscopy).

The objective of the current study was to determine the sensitivity and specificity of the Hologic Aptima BV and CV/TV assays relative to diagnostic SOC methodology in women with symptoms of vaginitis in the UK using the Hay/Ison criteria for BV detection by microscopy and microbial culture for *Candida* species.

## Methods

### Study design

This was a cross-sectional, diagnostic accuracy study conducted to evaluate the performance of the Aptima BV and Aptima CV/TV assays (Hologic Inc., San Diego, CA, USA) for the diagnosis of infectious vaginitis, relative to microscopy, culture and sensitivity methodology (referred to as SOC throughout). Aptima and SOC vaginitis testing were performed on vaginal swab samples from women with symptoms of vaginitis attending primary care centres served by the Severn Infection Sciences Laboratory, UK Health Security Agency, Bristol, UK. The Aptima Vaginitis panels are performed using vaginal swabs collected using the Aptima Multitest Swab Specimen Collection Kit (Hologic), whereas SOC tests are performed using swabs in Liquid Amies medium. For this study, remnant Aptima swabs from women aged ≥18 years undergoing *Chlamydia trachomatis* and *Neisseria gonorrhoeae* testing using the Aptima^®^ Combo 2 (AC2) assay (Hologic Inc., San Diego, CA, USA), alongside SOC testing for vaginitis, were used for Aptima BV and CV/TV testing. Exclusion criteria included women <18 years of age and cases where SOC or AC2 swabs were either not received or unavailable for testing.

### Sample identification

A weekly search of the North Bristol Trust Laboratory Information System was performed to identify eligible samples: explicitly, remnant swabs from AC2 assays from the preceding 7 days from women also undergoing SOC testing for the investigation of infectious vaginitis. Eligible samples were collected between 12 March and 17 September 2024.

### Diagnostic SOC tests

For BV, swabs for SOC testing underwent routine Gram stain and microscopy and were assessed using Hay/Ison criteria [22]. *Candida* species were identified by standard culture methods. *T. vaginalis* was not routinely tested for by culture or microscopy as TV has a low prevalence (<1.0%) in the patient population served by this laboratory. However, when requested by the submitting physician, testing was performed using the Aptima TV assay (Hologic).

### Aptima assays

The AC2 assay is designed for dual detection of *Chlamydia trachomatis* and *N. gonorrhoeae.* The Aptima BV assay detects the relative abundance of *Lactobacillus* species, *Atopobium vaginae* and *Gardnerella vaginalis*. The instrument software interprets the result, reporting the absence of BV in samples with a predominance of *Lactobacillus* species, and the presence of BV in samples where *Lactobacillus* species are depleted and replaced by *A. vaginae* or *G. vaginalis*. The multiplex Aptima CV/TV assay detects *Candida* species (combined detection of *Candida albicans*, *Candida tropicalis*, *Candida parapsilosis* and *Candida dubliniensis*), with separate detection of *C. glabrata* and *T. vaginalis*. A standalone Aptima TV assay, which solely identifies the presence of *T. vaginalis*, is also available. Where performed, this was utilized for comparison against Aptima CV/TV.

Each of the aforementioned assays uses multiplex transcription-mediated amplification to detect and (in some cases) quantify ribosomal RNA from indicator microorganisms in vaginal swabs using the Hologic Panther^®^ system, as per the manufacturer’s recommendations (Hologic Inc., San Diego, CA, USA). Vaginal swabs were collected using the Aptima^®^ Multitest Swab Specimen Collection Kit (Hologic Inc., San Diego, CA, USA) in accordance with the manufacturer’s instructions. Samples were loaded onto the Hologic Panther system and assigned to the appropriate assay via the system’s user interface for fully automated analysis.

### Confirmatory testing and discrepancy analyses

Selected samples showing concordant or discrepant results between SOC and Aptima assays for the detection of BV or CV were further analysed with real-time PCR using the TrueMark^™^ Vaginitis Plus Panel, a Pan-*Candida* assay and a *C. glabrata* assay (Thermo Fisher Scientific Inc., Waltham, MA, USA). The Vaginitis Plus Panel includes PCR assays for the detection of Pan-*Lactobacillus* (including *Lactobacillus jensenii*, *Lactobacillus iners*, *Lactobacillus gasseri* and *Lactobacillus crispatus*), *G. vaginalis*, *A. vaginae*, BV-associated bacteria 2, *Bacteroides fragilis*, *Megasphaera* type 1, *Mobiluncus curtisii*, *Prevotella bivia* and RNase P (endogenous human DNA control). The Pan-*Candida* assay includes real-time PCR assays for *C. glabrata*, *C. parapsilosis*, *C. tropicalis* and *C. albicans*. Assays were performed on the QuantStudio platform according to the manufacturer’s protocol (Thermo Fisher Scientific Inc., Waltham, MA, USA). Using cycle threshold (C*t*) values as a semi-quantitative measure of bacterial load, we compared bacterial loads in patients with and without BV or CV, as diagnosed by Aptima assay or SOC. As the TrueMark panel assays are intended for research use only, and not for use in clinical diagnostics, this discrepancy analysis for the detection of BV or CV should be considered exploratory in nature.

Additional analyses were performed to evaluate the concordance of the Aptima multiplex CV/TV assay vs. the standalone Aptima TV assay for the detection of *T. vaginalis* in order to support the use of the combined assay for the purpose of this study.

### Statistical analysis

Analyses of a paired sample dataset (comprising samples with both SOC and Aptima assay data) were undertaken to determine the sensitivity, specificity, positive and negative predictive values and diagnostic accuracy of the Aptima BV and Aptima CV/TV assays, relative to SOC diagnostic methods. These parameters were calculated using the MedCalc Diagnostic test evaluation online calculator [42].

Comparison of mean C*t* values from TrueMark Vaginal Plus Panels, used for confirmatory testing and discrepancy analysis, was performed by Student’s t-test. For the purpose of statistical analysis and graphical representation, PCR-negative results were assigned a notional C*t* value of 45.

### Ethics approval

This was a diagnostic accuracy study using routinely collected, fully anonymized, remnant diagnostic samples. Furthermore, patients whose samples were used were not enrolled in the study, nor were their samples stored for longer than the duration of time needed to ensure that all required routine laboratory testing had been completed (up to 60 days). Therefore, additional informed consent and research ethics approval were not required for this study, as verified by the NHS Health Research Authority’s online ethics approval tool.

## Results

### Study population and baseline demographics

**Fig. 1.**
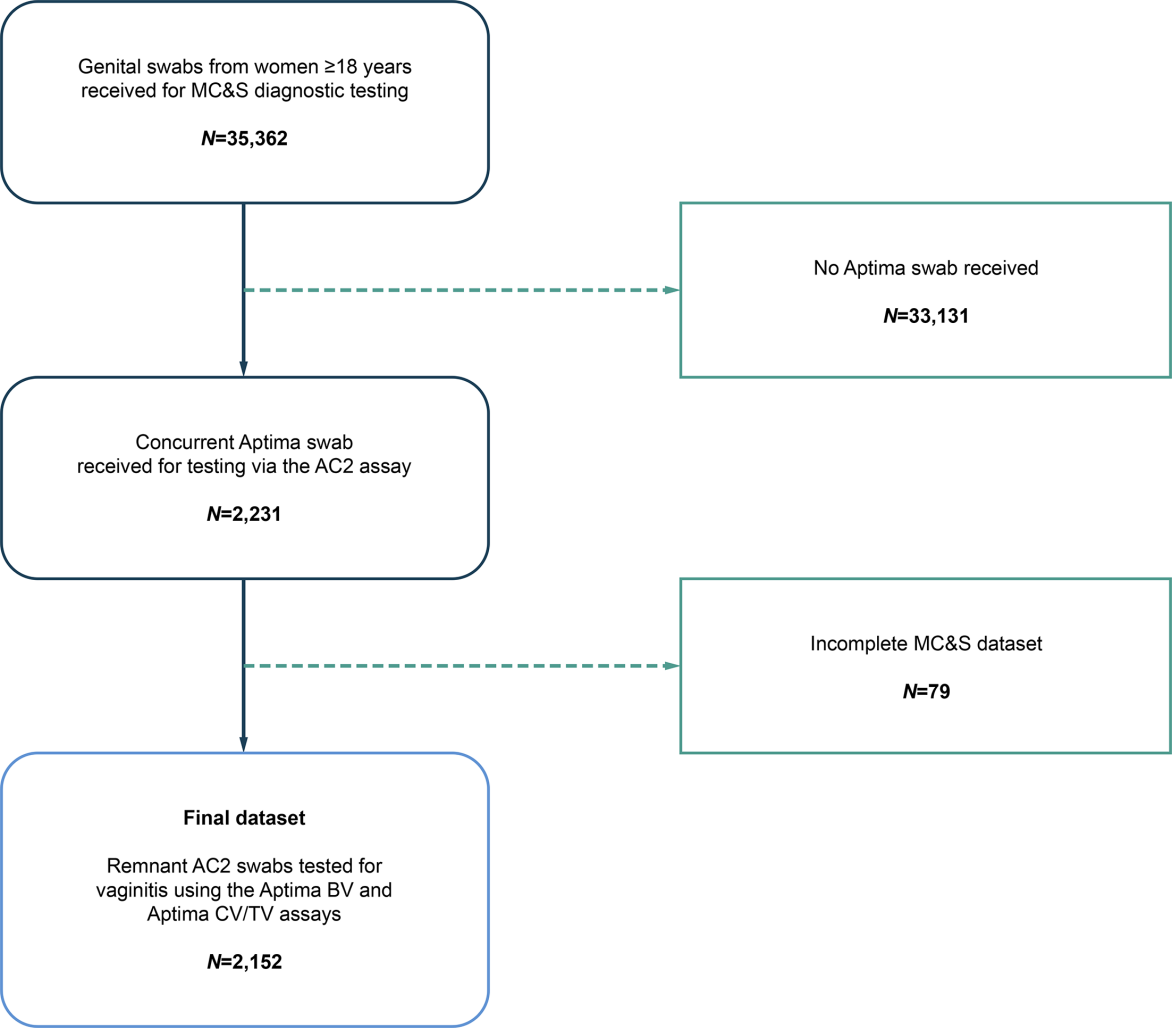
Patient and sample flow diagram. MC and S, microscopy, culture and sensitivity. The final paired dataset comprised remnant vaginal swabs from 2,231 women, which were taken for chlamydia and gonorrhoea testing via the AC2 testing. Each paired remnant swab (*N*=2,231) was tested for vaginitis via SOC methods in concurrence with testing via the Aptima BV and Aptima CV/TV assays.

Genital swabs from 35,362 women were received for SOC diagnostic testing for vaginitis during the study period; 2,231 of these women had concurrent swabs taken for chlamydia and gonorrhoea testing using the AC2 assay. From this total, 2,152 remnant AC2 swabs were then tested for vaginitis using the Aptima BV and Aptima CV/TV assays (Fig. 1). The mean (standard deviation) age of individuals undergoing routine testing for vaginitis was 35.1 (11.5) years.

### Infection rates

Vaginitis infection rates based on SOC methods and Aptima testing are shown in Tables 1 and 2, respectively. Overall, using SOC methods, BV (Hay/Ison grade 3) was detected in 11.4% of samples, while 5.6% were classed as intermediate (grade 2). *Candida* species were cultured in 25.6% of samples (Table 1). Using the Aptima vaginitis assays, BV was detected in 34.0% of samples, CV in 31.3%, *C. glabrata* in 1.9% and TV in 1.0% (Table 2).

**Table 1.** Vaginitis infection rates determined by SOC microscopy and culture*

Infection	Infection rate, *n* (%) *N*=2,152
BV (Hay/Ison criteria)	
Grade 1 (normal)	1,785 (82.9)
Grade 2 (intermediate)	120 (5.6)
Grade 3 (BV-positive)	247 (11.4)
CV	551 (25.6)
TV^b^	8 (10.7)†
All negative	1,309 (60.8)
**Coinfection**	
Grade 3 BV/CV	44 (2.0)

* The paired sample dataset comprised samples evaluated with both SOC methodology and Aptima assays; †TV has a low prevalence in our patient population; therefore, *T. vaginalis* was not routinely tested for by culture or microscopy. Samples were tested by Aptima TV assay only on clinician request (*n*=75); therefore, TV infection rates were calculated using 75 as the denominator.

N, total number of evaluable samples; n, number of infection-positive samples.

**Table 2.** Vaginitis infection rates determined by Aptima vaginitis assays*

Assay	Assay result	Infection rate, *n* (%) *N*=2,152
**Aptima BV**	Positive	732 (34.0)
	Negative	1,402 (65.1)
	Invalid	18 (0.8)
**Aptima CV/TV**		
*Candida* species	Positive	673 (31.3)
	Negative	1,444 (67.1)
	Invalid	35 (1.6)
*C. glabrata*	Positive	40 (1.9)
	Negative	2,077 (96.5)
	Invalid	35 (1.6)
*T. vaginalis*	Positive	22 (1.0)
	Negative	2,095 (97.4)
	Invalid	35 (1.6)
	**Coinfection**	
	BV/CV	245 (11.4)
	BV/TV	10 (0.5)
	BV/CV/TV	2 (0.1)
	*Candida* species/*C. glabrata*	10 (0.5)
	**All negative**	933 (43.4)

* The paired sample dataset comprised samples tested with both the SOC methodology and the Aptima assays.

N, total number of evaluable samples; n, number of infection-positive samples.

The proportion of samples that tested negative for infectious vaginitis was 60.8% using SOC methods (Table 1), compared with 43.4% using Aptima vaginitis assays (Table 2). These data indicate that a substantially higher number of patients would have received a positive diagnosis of infectious vaginitis based on results from the Aptima assays vs. SOC methods.

Results from the AC2 assay were available for 2,150 out of 2,152 samples and indicated that chlamydia and gonorrhoea were detected in 1.2% (*n*=26/2,150) and 0.1% (*n*=3/2,150) of women in this study, respectively.

### Performance of Aptima BV and Aptima CV/TV assays

The performance data for the Aptima BV and Aptima CV/TV assays relative to SOC methods are shown in Table 3. The Aptima CV/TV performance data are based on the detection of the *Candida* species group and/or *C. glabrata*, since culture does not differentiate *C. glabrata* from other species. Sensitivity and specificity estimates were 98.8% and 77.7% for Aptima BV and 94.9% and 88.3% for Aptima CV/TV, respectively. Positive and negative predictive values were 37.5% and 99.8% for Aptima BV and 74.0% and 98.0% for Aptima CV/TV, respectively. Diagnostic accuracy values were 80.2% for Aptima BV and 90.0% for Aptima CV/TV. Notably, of the 120 samples classified as intermediate (grade 2) by Hay/Ison criteria, 98 (81.7%) tested BV-positive using the Aptima BV assay.

**Table 3.** Clinical performance of Aptima BV and CV/TV assays relative to SOC methods*

Assay	Samples, *n*	Sensitivity, % (95% CI)	Specificity, % (95% CI)	Positive predictive value, % (95% CI)	Negative predictive value, % (95% CI)	Accuracy, % (95% CI)
**Aptima BV†**	2015	98.8 (96.4, 99.7)	77.7 (75.7, 79.6)	37.5 (35.5, 39.6)	99.8 (99.3, 99.9)	80.2 (78.4, 81.9)
**Aptima CV/TV‡, §**	2117	94.9 (92.7, 96.6)	88.3 (86.6, 89.8)	74.0 (71.2, 76.5)	98.0 (97.2, 98.6)	90.0 (88.6, 91.2)

* The paired sample dataset comprised samples tested with both SOC methodology and Aptima assays.

†Samples with grade 2 (intermediate) microscopy for BV or invalid Aptima BV results were excluded from this analysis.

‡The Aptima CV/TV performance was based on detection of the *Candida* species and/or *C. glabrata* group, since culture does not differentiate between these species. Samples with invalid Aptima CV/TV results were excluded from this analysis.

§It was not possible to determine the clinical performance of the Aptima CV/TV assay for TV relative to SOC methods, as TV was not routinely tested for by culture or microscopy.

CI, confidence interval.

### Confirmatory testing and discrepancy analysis

To further test the diagnostic accuracy of the Aptima vaginitis assays, a series of additional exploratory analyses using the real-time PCR TrueMark Vaginal Plus Panels was performed on selected samples from 139 women representing different categories of concordant and discordant Aptima BV and Hays Microscopy findings. These included those with (i) positive Aptima BV results and abnormal (Hays grade 3) microscopy (A^+^/M^+^; *n*=37); (ii) positive Aptima BV results and intermediate (Hays grade 2) microscopy (A^+^/M^eq^; *n*=16); (iii) positive Aptima BV results and normal (Hays grade 1) microscopy (A^+^/M^-^; *n*=41); and (iv) negative Aptima BV results and normal (Hays grade 1) microscopy (A^-^/M^-^; *n*=43). Two sample pairs were excluded from this analysis: one that tested invalid using the Aptima BV assay with abnormal microscopy and one that was Aptima BV-negative with intermediate microscopy.

As part of our confirmatory analysis, we compared bacterial loads in patients with and without BV as diagnosed by Aptima BV or SOC, using C*t* values from the TrueMark Vaginal Plus assays as a semi-quantitative measure of bacterial load (where the C*t* value is inversely related to bacterial load). Summary results for this analysis are shown in Table 4. Results of *B. fragilis* are not shown and were not included in the analyses because only 3 out of 137 samples tested positive for this organism. When using the Aptima BV assay for diagnosis, BV-positive samples had significantly lower mean levels of *Lactobacillus* species than samples deemed BV-negative via Aptima BV (mean C*t* value: BV-negative, 23.9; BV-positive, 29.2; *P*=0.0004; Table 4; row a, column *i*). This finding was not observed when BV was diagnosed by SOC [mean Ct value: grade 1 (normal), 28.0; grade 3 (abnormal), 25.5; *P*=0.1154; Table 4; row a, column *ii*]. In contrast, the other six BV-associated bacterial species assessed were present at significantly higher mean levels in patients with a positive BV diagnosis than in those with a negative diagnosis, whether assessed by Aptima BV (Table 4; rows b–g, column *i*) or SOC (Table 4; rows b–g, column *ii*). The RNase P assay assesses the human cellular content of the sample and is intended to act as a sample quality indicator. We observed a significant increase in mean RNase P in samples from women diagnosed with BV by SOC compared with those who tested negative (Table 4; row h, column *ii)*, an observation that was not found when using the Aptima BV assay for diagnosis (Table 4; row h, column *i*).

**Table 4.** Bacterial load of representative BV-associated bacteria in vaginal swabs from women with and without BV diagnosed by Aptima BV and Hay’s microscopy

		(i) Aptima BV result		(ii) Hays microscopy grade	
		Negative	Positive	Grade 1 (normal)	Grade 3 (abnormal)
**Thermo Fisher PCR assay**	***N* samples**	43	94	84	37
a) Pan-*Lactobacillus*	**Mean C*****t*** **value**	**23.9**	**29.1**	**28.0**	**25.5**
	sd	7.3	8.0	9.0	5.5
	sem	1.1	0.8	1.0	0.9
	***P-*** **value**	**0.0004**		**0.1154**	
b) *G. vaginalis*	**Mean C*t* value**	**36.7**	**19.0**	**29.3**	**15.8**
	sd	9.1	6.8	11.0	5.4
	sem	1.4	0.7	1.2	0.9
	***P*-value**	**<0.0001**		**<0.0001**	
c) *A. vaginae*	**Mean C*t* value**	**43.1**	**25.9**	**37.1**	**21.0**
	sd	3.9	9.8	9.9	6.3
	sem	0.6	1.0	1.1	1.0
	***P*-value**	**<0.0001**		**<0.0001**	
d) BV-associated bacteria 2	**Mean C*t* value**	**41.6**	**26.2**	**37.9**	**16.9**
	sd	5.9	14.6	9.6	12.4
	sem	0.9	1.5	1.0	2.0
	***P*-value**	**<0.0001**		**<0.0001**	
e) *Megasphaera* type 1	**Mean C*t* value**	**44.3**	**28.6**	**41.3**	**17.7**
	sd	2.8	16.0	8.7	14.3
	sem	0.4	1.7	0.9	2.4
	***P*-value**	**<0.0001**		**0.0001**	
f) *Mobiluncus curtisii*	**Mean C*t* value**	**45.0**	**39.2**	**43.6**	**34.3**
	sd	0.0	8.2	4.6	10.5
	sen	0.0	0.8	0.5	1.7
	***P*-value**	**<0.0001**		**<0.0001**	
g) *Prevotella bivia*	**Mean C*t* value**	**35.8**	**29.3**	**33.0**	**27.5**
	sd	7.5	7.8	7.5	8.1
	sem	1.1	0.8	0.8	1.3
	***P*-value**	**<0.0001**		**0.0004**	
h) RNAse P	**Mean C*t* value**	**25.3**	**24.4**	**25.6**	**21.8**
	sd	2.6	4.0	2.9	2.9
	sem	0.4	0.4	0.3	0.5
	***P* value**	**0.1547**		**<0.0001**	

C*t* values from the Thermo Fisher TrueMark Vaginal Plus PCR assays were used as a semi-quantitative measure of bacterial load (where C*t* value is inversely related to bacterial load) in patients with and without BV, as diagnosed by the Aptima BV assay or SOC (Hays/Ison microscopy).

**Fig. 2.**
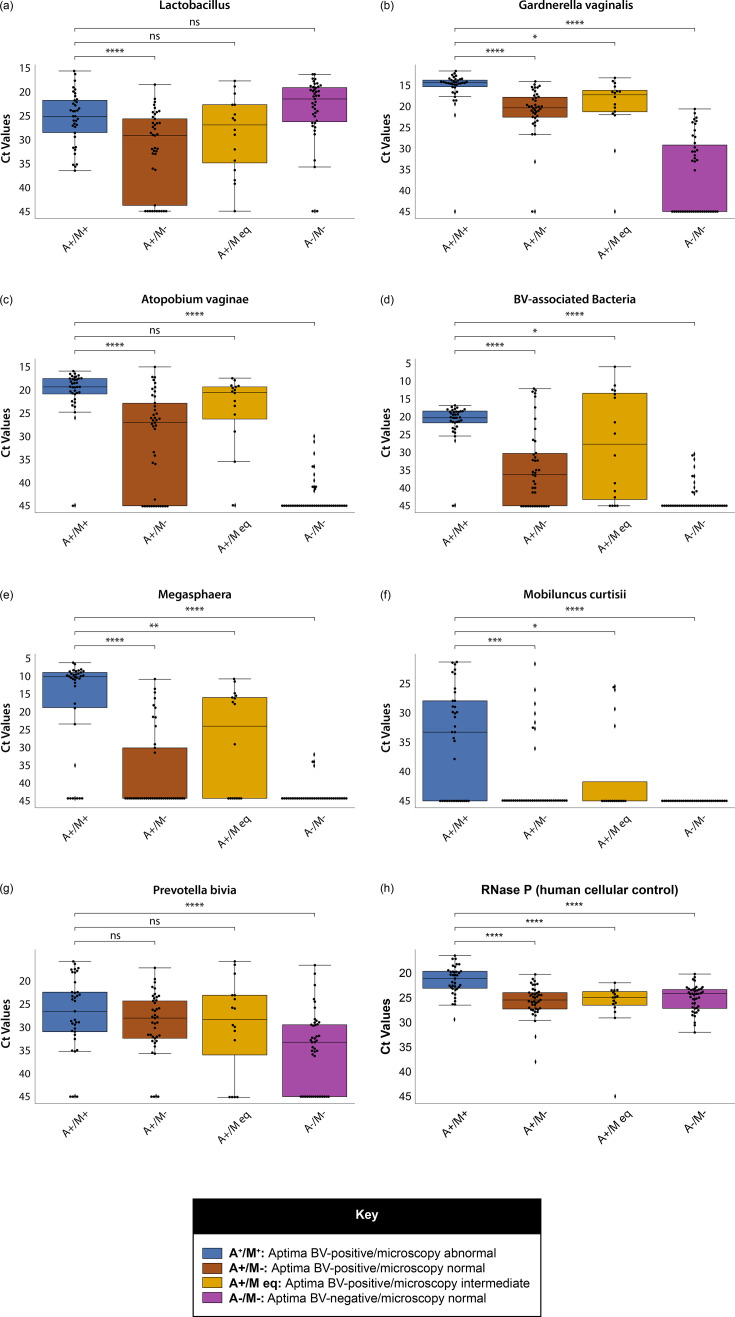
BV-associated bacterial loads in samples from women with concordant and discordant findings by Aptima BV and Hays microscopy. ns, not significant; *P<0.05; **P<0.005; ***P<0.0005; ****P<0.00005. *C*t values from the Thermo Fisher TrueMark Vaginal Plus PCR assays were used as a semi-quantitative measure of bacterial load in patients with and without BV, as diagnosed by the Aptima BV assay or SOC (Hays/Ison microscopy).

Regarding the BV discrepancy analysis, *Lactobacillus* levels were significantly lower in concordant BV-positive samples (A^+^/M^+^), compared to discordant samples that were positive only by Aptima BV (A^+^/M^-^). Across all other comparators (A^+^/M^eq^ and A^-^/M^-^), there were no significant differences in *Lactobacillus* levels (Fig. 2a). In contrast, analysis of other BV-associated markers showed that samples from women with concordant BV-positive results (A^+^/M^+^) had microflora characterized by significantly higher levels of several BV-associated bacteria when compared to those with concordant BV-negative results (A^-^/M^-^; *P*<0.001, Fig. 2b–g). Samples from women who tested Aptima BV positive but had normal or intermediate findings by microscopy (A^+^/M^-^ and A^+^/M^eq^) typically had intermediate levels of other BV-associated bacteria relative to those with concordant positive or negative BV results (Fig. 2b–g). Although RNase P levels showed little variation across sample groups, samples from patients who were BV-positive by microscopy had significantly higher RNase P levels than those classed as BV-negative or intermediate by microscopy, as previously noted above (Fig. 2h).

The subset of 139 sample pairs described above was also used for confirmatory and discrepancy analysis of *Candida* detection by SOC and Aptima CV/TV, using the Thermo Fisher Pan-*Candida* and *C. glabrata* assays. Two sample pairs, which tested invalid using the Aptima CV/TV assay, were excluded from this analysis. Among this cohort, the Aptima CV/TV assay showed 97.6% sensitivity and 86.5% specificity, relative to the Pan-*Candida* and *C. glabrata* assays (Fig. S1, available in the online Supplementary Material). Culture methods for the detection of *Candida* species showed 85.4% sensitivity and 99.0% specificity, relative to the Pan-*Candida* assay (Fig. S2). Based on analysis of seventeen discrepant samples, the *Candida* PCR assays supported a *Candida*-positive result in 4 out of 16 samples, which were *Candida*-positive by Aptima CV/TV but culture negative, and in one additional sample that was *C. glabrata*-positive by Aptima CV/TV but culture-negative (Table S1). Across this cohort, 100% concordance was observed between the *C. glabrata*-specific TrueMark assays vs. the Aptima CV/TV assay for the detection of *C. glabrata* (Table S2).

Regarding use of the combined multiplex Aptima CV/TV assay in this study, as opposed to the standalone Aptima TV assay, where TV testing was requested by the clinician (*n*=75), the Aptima CV/TV assay demonstrated sensitivity of 87.5% and specificity of 100% for the detection of TV, relative to the standalone Aptima TV assay (Table S3).

## Discussion

We set out to evaluate the performance of the Aptima BV and Aptima CV/TV assays, relative to SOC methodology, for the diagnosis of infectious vaginitis in women attending primary care centres in Southwest England. In addition to offering high analytical sensitivity and specificity for the detection of infectious causes of vaginitis, the Aptima vaginitis assays offer additional benefits that may help to overcome many of the logistical challenges associated with current SOC methods, such as loss of sample quality during transit, high staffing requirement for manual processing, reliance on skilled personnel for slide reading and subjective interpretation, which make SOC less suitable for streamlined, large-scale clinical implementation. The automated, multiplex capabilities of the Hologic Panther system and the Aptima Vaginitis assays allow for differential diagnoses of BV, CV and TV from a single vaginal swab in just a few hours, unaffected by coinfection status. In addition, the ability to distinguish *C. glabrata* from other *Candida* species is helpful in guiding appropriate treatment for *C. glabrata*, which is fluconazole-resistant.

Using SOC microscopy, BV (Hay/Ison Grade 3) was observed in 11.4% of women, whereas the Aptima BV assay detected BV in 34.0% of our cohort. The *Candida* species group was present in 25.6% of women by culture, compared with 31.3% by Aptima CV/TV assay, including 1.9% in whom fluconazole-resistant *C. glabrata* (recently renamed as *Nakaseomyces glabrata* [43]) was detected. In line with the low expected prevalence in this cohort, *T. vaginalis* was detected in 1.0% of women by Aptima CV/TV assay. The infection rates reported in our study are similar to those of previous Aptima BV and Aptima CV/TV evaluation studies, which reported rates of 28.6–38.2% for BV, 14.2–32.8% for CV and 1.0–3.0% for TV [33,39]. These findings suggest comparable Aptima vaginitis assay performance and the relative consistency of infection rates across different studies. Furthermore, the substantially higher detection rate of BV and CV using the Aptima assays vs. SOC methods in multiple studies suggests a greater diagnostic sensitivity associated with Aptima assays than conventional methods. The higher sensitivity of the molecular tests is a likely explanation for the low specificity values when less sensitive SOC tests are used as the comparator for statistical analyses. The results of discrepancy analysis in the present study support this conclusion in part; however, the possibility that the Aptima tests give false positive results or detect BV or CV which is not clinically relevant, requires further evaluation in studies that correlate microbiological findings with clinical presentations and outcomes.

In the present analysis, sensitivity and specificity values were 98.8% and 77.7% for the Aptima BV assay and 94.9% and 88.3% for the Aptima CV/TV assay, respectively, relative to SOC. These results were reflected in the overall diagnostic accuracy values, which were 80.2% for Aptima BV and 90.0% for Aptima CV/TV. For the detection of *Candida* species, our sensitivity and specificity findings were broadly in line with those of previous evaluation studies, which reported a sensitivity of 91.7–96.3% and a specificity of 85.6–94.9% with the Aptima CV/TV assay [29,33, 39]. However, while the sensitivity of the Aptima BV assay in the current study (98.8%) was consistent with previous reports of 90.0–98.4%, the specificity (77.7%) was lower than previous estimates, which reported values of 89.6–96.3% when samples with intermediate microscopy were excluded from the analysis [29,33, 38, 39]. This observation may reflect differences in the performance of microscopy with Hay/Ison criteria, as was used in this study, compared to microscopy with Nugent scoring, used in other studies.

Similarly, the positive predictive value of the Aptima BV assay for the detection of BV in our study was somewhat lower than previously published reports where Nugent scoring, as opposed to Hay/Ison criteria, was used as the SOC comparator (37.5% vs. 83.7%) [38]. This is likely due to the lower diagnostic sensitivity of Hay/Ison criteria.

These findings collectively underscore how differences in diagnostic reference standards can impact performance estimates of molecular assays, such as the Aptima Vaginitis assays. Gram-stain-based methodologies for the detection of BV, such as Hay/Ison or Nugent scoring, can also introduce ambiguity and diagnostic uncertainty, particularly in cases classified as ‘intermediate’. The clinical handling of intermediate BV results is an important consideration for patient management. A study by Taylor-Robinson *et al.* reported that only 37.2% (*n*=35/94) of cases classed as Hay/Ison grade 2 (intermediate) correlated with the clinical criteria for BV, compared with 91.7% (*n*=388) of grade 3 (BV-positive) reads [44]. In addition, approximately half (47.0%) of the women with grade 2/intermediate status in the aforementioned study failed to respond to clindamycin treatment. Collectively, these data suggest that a substantial proportion of women who were classed as grade 2 were likely to be BV-negative [44]. In contrast to the Taylor–Robinson study, we found that a high proportion (81.7%) of our Hay/Ison grade 2 samples were BV-positive by Aptima BV assay. This figure is in good agreement with that of Caza *et al.* [29], who reported that 85.7% of samples classed as intermediate based on Nugent scoring were deemed BV-positive by the Aptima BV assay. Overall, these findings suggest that the categorization of BV status as intermediate is limited by the inherently subjective nature of microscopy-based methods, as well as variation between scoring criteria, readers and testing centres. This highlights one of the key advantages of molecular testing over current SOC methods, namely that molecular diagnostic assays eliminate the intermediate/indeterminate category, providing objective, quantitative diagnostic information that is reproducible and amenable to standardization [31,32].

Our exploratory, confirmatory and discrepancy analyses using the Thermo Fisher TrueMark Vaginal Plus Panel largely corroborated the microbiological basis of both Aptima BV testing and Hays microscopy. Samples from women in whom Aptima BV testing was positive, but Hays microscopy was graded as 1 or 2 (normal or intermediate), had a microbial profile that was intermediate relative to concordant Aptima BV and microscopy results. This observation suggests that Aptima BV may identify emerging dysbiosis before it becomes microscopically evident.

Our observation of increased RNase P, indicative of increased mean cellularity, in samples from women diagnosed with BV by Hays microscopy may be a chance finding. However, it is possible that this observation may be due to the presence of clue cells, typically associated with BV, and a positive microscopic diagnosis [45].

As the TrueMark Vaginal Plus Panel is not validated for BV diagnosis and does not define quantitative diagnostic thresholds, this analysis cannot be used to arbitrate between discrepant results of Aptima BV and Hays microscopy. Furthermore, we observed some considerable variability within groups, which, in conjunction with a relatively low sample size, limits our ability to make any further conclusions. Nonetheless, these findings do suggest that samples with Aptima BV-positive results from women with normal or intermediate microscopy represent an intermediate status on a spectrum of microbial dysbiosis. Further evaluation against clinical endpoints may be required to understand the significance of these findings, and the use of microbial profiling, such as that provided by the TrueMark Vaginal Plus panel, may prove useful in such analyses.

The discrepancy analysis of *Candida* results suggests that discrepancies between the three tests (culture, Aptima CV/TV and Thermo Fisher *Candida* PCR assays) reflect differences in the relative sensitivity and specificity of each assay. The 100% concordance between the *C. glabrata*-specific TrueMark assay and the Aptima CV/TV assay indicates high specificity of Aptima CV/TV for *C. glabrata* detection.

As discussed above, although the Thermo Fisher TrueMark assays used in this study are intended for research use only and are not clinically validated for diagnostic purposes, they are considered to be analytically robust and widely used for microbial profiling. As such, these findings offer supportive evidence for the high sensitivity and specificity of the Aptima assays, though further comparison with clinically validated molecular tests is warranted.

This study is subject to a number of limitations. Firstly, it was not possible to determine the true clinical performance of the Aptima CV/TV assay for *T. vaginalis* relative to SOC methods, since *T. vaginalis* was not tested for by culture or microscopy due to low prevalence within our study population. Secondly, no clinical endpoint data, in terms of treatment or outcomes, were available for patients included in the study. To determine the clinical sensitivity and specificity of laboratory methods that give a microbiological diagnosis of vaginitis, further evaluation relative to clinically relevant outcomes will be required, as discussed above. Thirdly, our study population consisted of women undergoing concurrent testing for vaginitis, chlamydia and gonorrhoea (from whom both Aptima and liquid Amies swabs were received). We observed that the mean age of this population was, perhaps not unexpectedly, lower than the mean age of women undergoing testing solely for vaginitis (35 years vs. 38 years; not shown). While this modest age difference is unlikely to have significantly influenced our findings, it may limit the extent to which results can be generalized to the wider population of women presenting with vaginitis in routine clinical practice.

Nonetheless, the data generated from this study provide further evidence that the Aptima BV and Aptima CV/TV assays are capable of providing objective and quantitative results with high sensitivity and specificity for the diagnosis of vaginitis, consistent with previous observations using these assays, and can be considered for use in laboratories using Hay/Ison criteria for microscopic diagnosis of BV.

## Conclusions

The Aptima BV and Aptima CV/TV assays demonstrated high sensitivity and specificity in diagnosing infectious vaginitis, relative to SOC methods, with diagnostic accuracy values of 80.2% for Aptima BV and 89.8% for Aptima CV/TV. The automated, multiplex capabilities of the Hologic Panther system and Aptima molecular assays allow for differential diagnoses of BV, CV and TV from a single vaginal swab, unaffected by coinfection status. A further key advantage of the Aptima vaginitis assays is that they eliminate the intermediate/indeterminate category of BV associated with SOC testing to provide objective, quantitative diagnostic information about the vaginal microbiota. Overall, the Aptima BV and Aptima CV/TV assays represent a robust and reliable alternative to conventional vaginitis testing. Further studies are required to correlate assay results with treatment and clinical outcomes.

## Supplementary material

10.1099/jmm.0.002163 Supplementary Material 1.

## Abbreviations

AC2: Aptima Combo 2 Assay
BV: bacterial vaginosis
C*t*: cycle threshold
CV: *Candida* vaginitis
HIV: human immunodeficiency virus
SOC: standard-of-care
TV: *Trichomonas* vaginitis
