# Erratum: Evaluation of the Hologic Aptima Vaginitis assays for microbiological diagnosis of vaginitis in women with abnormal vaginal discharge attending primary care

**DOI:** 10.1099/jmm.0.002185

**Published:** 2026-07-08

**Authors:** Barry Vipond, Nicola Childs, Rich Hopes, David Hirst, Kshitij Soni, Paul North, Peter Muir

**Affiliations:** 1 UK Health Security Agency Clinical Network Laboratory, Southmead Hospital, Bristol, UK; 2 North Bristol NHS Trust, Southmead Hospital, Bristol, UK

During the peer review and production process of the original version of this article the Microbiology Society failed to request a conflict of interest statement reflecting the role of the research funders in the preparation of this article. The authors have kindly provided the following statement:

**Conflicts of interest**

Hologic commissioned the Infection Sciences Department at UKHSA Bristol to design and undertake an evaluation of the Aptima Vaginitis assays, and agreed to fund the study according to the sample size proposed by the laboratory. Hologic played no further role in the design of the study, analysis of data or the drafting and approval of the manuscript. The authors declare that there are no additional conflicts of interest.

The Microbiology Society apologise for any inconvenience caused.

