# Supplementary material for: Evaluation of the Hologic Aptima Vaginitis assays for microbiological diagnosis of vaginitis in women with abnormal vaginal discharge attending primary care
Source: J Med Microbiol. 2026 May 19;75(5):002163. doi: 10.1099/jmm.0.002163 (PMC13186402; doi:10.1099/jmm.0.002163)
Supplement: Supplementary Material 1. [file jmm-75-02163-s001.pdf]

Supplementary Figure 1: Confirmatory analysis of *Candida* detection by the Aptima CV/TV assay versus *Candida* PCR assays

		Aptima CV		
		Positive	Negative	Total
**Thermo Fisher *Candida* PCR**	Positive	40	13	53
	Negative	1	83	84
	Total	41	96	137

**Aptima CV performance**	**Value**	**95% CI**
Sensitivity	97.56	87.14-99.94
Specificity	86.46	77-96-92.59
Positive Predictive Value	75.47	64.93-83.64
Negative Predicitive Value	98.81	92.28-99.83
Diagnsotic Accuracy	89.78	83.45-94.30

The performance of the Aptima CV/TV assay was further evaluated against the Thermo Fisher *Candida* PCR assays (Pan-*Candida* and C. glabrata-specific assays). The above analyses were calculated using MedCalc's diagnostic test evaluation calculator.

Supplementary Figure 2. Confirmatory analysis of *Candida* detection by culture methods versus *Candida* PCR assays

		***Candida* culture**		
		Positive	Negative	Total
**Thermo Fisher *Candida* PCR**	Positive	35	1	36
	Negative	6	95	101
	Total	41	96	137

***Candida* culture performance**	**Value**	**95% CI**
Sensitivity	85.37	70.83-94.43
Specificity	98.96	94.33-99.97
Positive Predictive Value	97.22	83.22-99.60
Negative Predicitive Value	94.06	88.31-97.07
Diagnsotic Accuracy	94.89	89.76-97.92

The performance of culture methods for the detection of *Candida* species was further evaluated against the Thermo Fisher Pan-*Candida* PCR assay. The above analyses were calculated using MedCalc’s diagnostic test evaluation calculator.

Supplementary Table 1. Discrepancy analysis of the Aptima CV/TV assay versus culture methods for the detection of *Candida* species

		Aptima CV/TV assay				
		***Candida* species positive only**	***C. glabrata* positive only**	**Dual *Candida* species/ *C. glabrata* positive**	**Negative**	**Total**
***Candida* culture**	**Positive**	29	5	2	0	36
	**Negative**	16*	1**	0	84	101
	**Total**	45	6	2	84	137

* 4/16 of these cases confirmed as *Candida*-positive by Pan-*Candida* PCR assay.

** Confirmed as *C. glabrata*-positive by *C. glabrata* PCR assay.

Supplementary Table 2: Concordance analysis of *C. glabrata* detection with Aptima CV/TV versus Thermo Fisher PCR[a] assays

Assay (Target)	Samples, n	Sensitivity, % (95% CI)	Specificity, % (95% CI)	Positive predictive value, % (95% CI)	Negative predictive value, % (95% CI)	Accuracy, % (95% CI)
Aptima CV/TV (*C. glabrata*)	137[b]	100.0 (63.1, 100.0)	100.0 (97.2, 100.0)	100.0 (63.1, 100.0)	100.0 (97.2, 100.0)	100.0 (97.3, 100.0)

CI, confidence interval; CV, *Candida* vaginitis; TV, *Trichomonas* vaginitis.

[a]TrueMark Vaginal Plus Panel for sexually transmitted infections and vaginal health.

[b]Two samples that gave invalid results using the Aptima CV/TV assay were excluded from this analysis.

Supplementary Table 3: Concordance analysis of *T. vaginalis* detection with the Aptima CV/TV assay, versus the Aptima TV assay

**Assay (Target)**	Samples, n	Sensitivity, % (95% CI)	Specificity, % (95% CI)	Positive predictive value, % (95% CI)	Negative predictive value, % (95% CI)	Accuracy, % (95% CI)
Aptima CV/TV (*T. vaginalis*)	75	87.5 (47.4, 99.7)	100.0 (94.6, 100.0)	100.0 (59.0, 100.0)	98.5 (91.5, 99.8)	98.7 (92.8, 100.0)

CI, confidence interval; CV, *Candida* vaginitis; TV, *Trichomonas* vaginitis.
Concordant analysis was performed to confirm the validity of using the multiplex Aptima CV/TV assay for the detection of *T. vaginalis* in the present study, rather than the standalone Aptima TV assay.
